# Modeling Host-Virus Interactions in Viral Infectious Diseases Using Stem-Cell-Derived Systems and CRISPR/Cas9 Technology

**DOI:** 10.3390/v11020124

**Published:** 2019-01-30

**Authors:** Jihoon Kim, Bon-Kyoung Koo, Ki-Jun Yoon

**Affiliations:** 1 Institute of Molecular Biotechnology of the Austrian Academy of Sciences (IMBA), Vienna Biocenter (VBC), Dr. Bohr-Gasse 3, 1030 Vienna, Austria; jihoon.kim@imba.oeaw.ac.at; 2 Department of Biological Sciences, Korea Advanced Institute of Science and Technology (KAIST), Daejeon 34141, Korea

**Keywords:** organoid, host-virus interactions, CRISPR/Cas9 genome editing, induced pluripotent stem cell, adult stem cell, modeling of viral pathogenesis

## Abstract

Pathologies induced by viral infections have undergone extensive study, with traditional model systems such as two-dimensional (2D) cell cultures and in vivo mouse models contributing greatly to our understanding of host-virus interactions. However, the technical limitations inherent in these systems have constrained efforts to more fully understand such interactions, leading to a search for alternative in vitro systems that accurately recreate in vivo physiology in order to advance the study of viral pathogenesis. Over the last decade, there have been significant technological advances that have allowed researchers to more accurately model the host environment when modeling viral pathogenesis in vitro, including induced pluripotent stem cells (iPSCs), adult stem-cell-derived organoid culture systems and CRISPR/Cas9-mediated genome editing. Such technological breakthroughs have ushered in a new era in the field of viral pathogenesis, where previously challenging questions have begun to be tackled. These include genome-wide analysis of host-virus crosstalk, identification of host factors critical for viral pathogenesis, and the study of viral pathogens that previously lacked a suitable platform, e.g., noroviruses, rotaviruses, enteroviruses, adenoviruses, and Zika virus. In this review, we will discuss recent advances in the study of viral pathogenesis and host-virus crosstalk arising from the use of iPSC, organoid, and CRISPR/Cas9 technologies.

## 1. Introduction

Viruses are obligatory intracellular pathogens that rely on host cell surface receptors to enter the cell, and co-opt host cellular machineries to replicate, assemble, and release new virus particles. On the other hand, host cells can also detect and counteract invasion by viruses by sensing pathogen-associated molecular patterns, such as nucleic acids of viral origin [1]. Although antiviral drugs targeting viral proteins have demonstrated efficacy, such activity is often hampered by the evolution of drug-resistance mechanisms, such as the rapid selection of mutations in the drug binding site [2,3,4]. By contrast, drugs targeting the host factors upon which the virus depends are far less susceptible to the rapid emergence of resistance. Thus, the identification of host factors that promote and restrict viral replication can provide both important insights into basic aspects of host-virus interaction and novel therapeutic targets for antiviral drug development.

Studies of viral pathogenesis have traditionally utilized in vitro culture systems of transformed human cell lines. Whilst transformed cell lines are simple and cost-effective model systems, they frequently fail to accurately recapitulate the characteristics and signaling pathways of normal human cells [5,6]. In addition, because most cell lines are composed of a homogeneous cell type, they are unsuitable for modeling the interactions among different cell types within the complex architecture of normal tissue. To address these limitations, there has been widespread use of animal models of viral pathogenesis, including rodent and primate models. However, many human viruses do not effectively infect or reproduce human pathophysiology in these animal models [7,8], limiting their utility for studying host-virus interaction and molecular mechanisms of human viral diseases.

**Figure 1:**
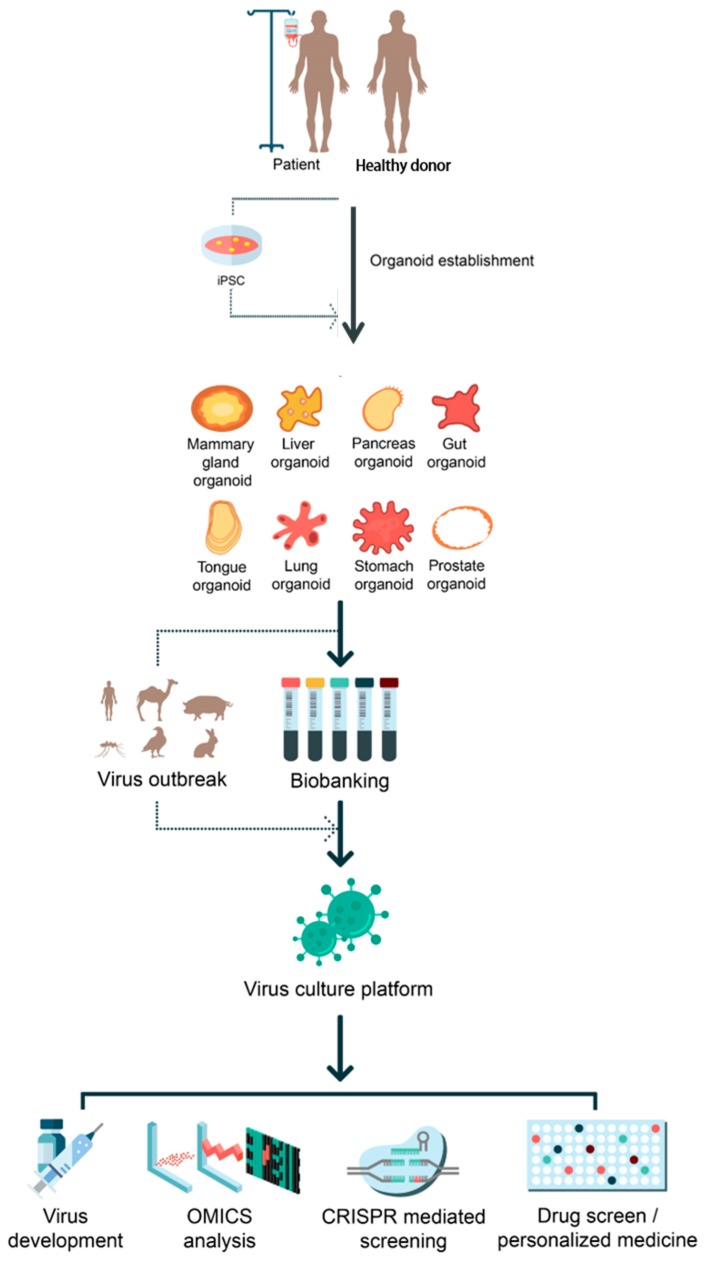
Applications of organoids on virus-mediated diseases. Potential applications of iPSC-derived and adult stem-cell-derived organoid systems for the viral pathogen outbreak are illustrated. Established organoids from different organs can serve as a proper virus culture platform for many purposes such as vaccine generation, OMICS analysis, CRISPR/Cas9-mediated screening, and drug screening for personalized medicine.

Several recent technological advances have provided human in vitro systems that more accurately recapitulate in vivo physiology and are readily amenable to virus research. First, the development of induced pluripotent stem cell (iPSC) technology has allowed unprecedented opportunities to study the pathobiology of human disorders. iPSC cells with embryonic stem cell features were first derived from mouse fibroblasts by ectopic expression of four stem cell transcription factors (i.e., Oct4, Sox2, Klf4, and c-Myc) [9]. In the following year, human iPSCs were generated from human somatic skin cells by using similar reprogramming protocols [10,11]. Like embryonic stem cells, human iPSCs can be grown and indefinitely differentiated into a variety of cells [12,13]. In last decades, iPSC-derived systems have been widely used in a variety of research on human diseases [14,15] Recently, iPSC technology has also provided versatile platforms for disease modeling, drug screening, and the investigation of host-virus interactions for virus researches. Patient-specific iPSC-derived systems enable the non-invasive establishment of systems to test novel antiviral therapies in an individualized setting, consistent with the principles of personalized medicine and without the ethical questions that surround embryonic stem cell systems. Second, rapid advances in generating three-dimensional (3D) organoid models from pluripotent stem cells or adult stem cells provide easily accessible and robust platforms amenable to genetic manipulation [16,17,18,19]. Third, development of CRISPR/Cas9 genome editing enables the rapid introduction of targeted mutations or genome-wide screening in stem-cell-derived systems [20,21,22]. The combination of these technologies thus provides versatile and patient-specific human model systems to study virus infection and pathogenesis [19,23,24,25,26]. Here we review recent progress in our understanding of host-virus interaction mechanisms in human viral diseases using human stem-cell-derived model systems, combined with CRISPR/Cas9 genome editing technology (Figure 1).

## 2. Modeling of Viral Disease Using iPSC Technology

Although a wealth of genome-wide association studies (GWAS) have implicated specific genes conferring increased susceptibility or resistance to specific infections [27,28,29], until recently only a limited number of in vitro model systems have been available to investigate the interplay between viral infection and host genetics. Patient-specific iPSC-derived systems have transformed the study of host-virus interactions. iPSC lines established from individuals with documented sensitivity or resistance to viral diseases can be utilized for the validation of host factors identified in GWAS datasets. This is especially relevant when we take into consideration that efficient and robust protocols are now available to generate the cell types of interest from iPSC, to model viral diseases in different tissues [30,31]. In addition to iPSC technology itself, the precise introduction of mutations into human iPSCs by CRISPR/Cas9 technology is advantageous in several ways. By introducing specific mutations into standardized iPSC lines, it is possible to bypass reprograming and biopsy procedures to reduce variability of iPSC lines and ethical issues. In addition, the resultant isogenic iPSC lines eliminate the variability arising from individual genetic backgrounds.

However, several limitations remain when modeling human tissues using traditional 2D cultures of differentiated cell types. It is largely impossible to reconstitute physiologically accurate gradients of patterning molecules, endogenous growth factors and nutrients in such culture systems. In addition, apical-basal polarity and normal cell-cell junctions are not maintained in monolayer cultures, and most cell types become flattened when cultured in 2D on plastic dishes. Indeed, 2D and 3D cultures often show different outcomes in drug screening [32,33]. Thus, the development of culture systems that recapitulate more complex cell–cell interactions and cell diversity, similar to normal human tissues, is crucial to study complex host-virus interactions in infectious diseases. Human organoids are 3D cultures derived from human pluripotent stem cells, adult stem cells or primary tissues. Organoids have the ability to self-renew and self-organize to develop organ-like structures that recapitulate the unique cytoarchitecture of primary tissues. Organoid systems have been described for multiple human organs, such as the brain, intestines, stomach, liver, lungs, pancreas, and retina [34]. Human iPSC-derived systems in both 2D and 3D have been actively used to study host-virus interactions for various viruses [35,36]. In this part, we review the recent application of human iPSC-derived systems to Zika virus research as an example of how such systems are used.

The recent Zika virus (ZIKV) outbreak in South America and its suspected link with neurological disorders, including microcephaly, led the World Health Organization to declare a global health emergency [37]. Microcephaly is characterized by smaller head circumference, intellectual disability, and seizures, and is due to reduced neuronal production or increased cell death from neural progenitor cells (NPCs) [38]. Although clinical examination of microcephalic fetal tissues showed the presence of ZIKV in the amniotic fluid and damaged fetal brains [39,40], a mechanistic understanding of how ZIKV induces damage during embryonic brain development was limited due to the variable quality and genetic background of clinical samples. Therefore, human stem-cell-derived models were adopted to study the cellular tropism and pathogenesis of ZIKV under controlled conditions.

The first in vitro study to model ZIKV infection of human neural cells utilized 2D cultures of iPSC-derived NPCs and neurons [41]. In this study, it was found that ZIKV preferentially infects NPCs over iPSCs or neurons, and results in increased cell death and dysregulated cell cycle progression. Another study showed that ZIKV also infects stem-cell-derived human neural crest cells and peripheral neurons in vitro, leading to increased cell death and transcriptional dysregulation [42]. These examples demonstrate the application of 2D human stem-cell-derived models to investigate virus tropism and cell type-specific pathology.

A number of studies using 3D human stem-cell-derived systems, including neurosphere culture and brain organoid models, revealed in detail the cellular phenotypes related to human microcephaly and neurological disorders that result upon ZIKV infection [18,43,44,45,46,47]. Given the complex organization of the developing brain, with the presence of multiple types of NPCs and neurons, 3D brain organoid models provide a much more physiologically relevant platform than 2D cultures to assess ZIKV pathogenesis. Crucially, the human developing cortex is composed of different NPCs with unique properties, such as ventricular radial glial cells (vRGCs), outer subventricular radial glial cells (oRGCs), and intermediate progenitor cells (IPCs), which are modeled by 3D brain organoids [48]. It was demonstrated that ZIKV exhibits tropism towards vRGCs and oRGCs over IPCs or immature neurons in human iPSC-derived 3D brain organoids [18]. Infected NPCs become viral factories, producing more infectious viral particles and leading to the propagation of ZIKV-infected cells throughout the 3D structure [18]. Meanwhile, intrinsic differences in the pathogenicity of different ZIKV stains have been extensively investigated using brain organoid models. It was found that ZIKV strains from Brazil produced more severe effects on neuronal layer thickness in human brain organoids when compared to an African strain [49]. A recent study showed two newly isolated strains of ZIKV, an American strain and a closely related Asian strain, productively infect human iPSC-derived brain organoids [46]. Interestingly, the main phenotypic effect of these two strains was the premature differentiation of neural progenitors associated with centrosome perturbation, which are different from those of an African strain, MR766.

Human stem-cell-derived models have also been extensively used to understand the underlying molecular pathways of host-virus interactions. First, genome-wide analyses of virus-infected models have proved a rich resource for unravelling host responses upon viral infection [41,45,47,50]. Transcriptome analysis of iPSC-derived, monolayer NPCs infected with different ZIKV strains revealed changes occurring in host gene expression during infection. Interestingly, in spite of the generally similar phenotypic impact of an Asian and an African strain, only the Asian strain induced the upregulation of TP53 and viral response genes in infected human NPCs [50]. Transcriptome analysis of ZIKV infection in human cerebral organoids identified the upregulation of toll-like receptor 3 (TLR3), an innate immune receptor, and further demonstrated that inhibition of TLR3 can ameliorate the effect of ZIKV infection [47]. Genome-wide methylome profiling in human ESC-derived brain organoids showed alterations in DNA methylation in neural progenitors, astrocytes and differentiated neurons at genes that have been implicated in the pathogenesis of a number of brain disorders [51], suggesting that ZIKV infection during fetal development could lead to a spectrum of delayed-onset neuropsychiatric complications. Second, interactions between viral proteins and host components have also been examined using human stem-cell-derived models. ZIKV produces three structural proteins (C, prM, and E) and seven nonstructural proteins (NS1, NS2A, NS2B, NS3, NS4A, NS4B, and NS5), but the interaction of these proteins with the host molecular machinery and the impaired neurogenesis were not well understood.

Using human fetal neural stem cells as a model system, one study found that the ZIKV proteins NS4A and NS4B inhibit the Akt-mTOR signaling pathway, disrupting neurogenesis and inducing autophagy [52]. Through systematic screening of ZIKV proteins, another study found that the ZIKV protein NS2A reduces neural stem cell proliferation by inducing the degradation of host adherent junction proteins in human forebrain organoids [43]. Finally, human stem-cell-derived models have been used for the screening and validation of therapeutic interventions against viruses. For example, a drug screening of over 6,000 compounds, including FDA-approved drugs and candidates in clinical trials, was performed in iPSC-derived NPC cultures to find compounds that prevent ZIKV-induced cell death. This study identified several potent hits, such as Emricasan, and proceeded to validate those hits using human forebrain organoids [53]. Another study has reported differing degrees of efficacy of various compounds in mitigating ZIKV-induced cytopathy in an optimized human brain organoid system [45]. In summary, 2D and 3D iPSC- and ESC-derived model systems have been applied in multiple ways to provide significant insight into the host factors, pathogenesis and underlying biological mechanisms involved in virus infection as well as platforms for drug testing.

iPSC-derived systems were also utilized for researches on other viruses. For example, iPSC-derived hepatocyte-like cells support the entire life cycle of the hepatitis C virus (HCV) and induced an antiviral inflammatory response as a versatile in vitro platform for HCV research [54,55].

On the other hand, human cytomegalovirus (HCMV) infection is one of the leading prenatal causes of congenital mental retardation and deformities world-wide [56], but difficulties of human primary neuronal culture have been major limitation to study HCMV. Thus, iPSCs have also used as models to investigate HCMV infection and cellular response of human fetal brain, revealing NPCs are fully permissive for HCMV infections and showed impairments of their differentiation potential upon HCMV infection [57]. In summary, 2D and 3D iPSC- and ESC-derived model systems have been applied in multiple ways to provide significant insight into the host factors, pathogenesis and underlying biological mechanisms involved in virus infection as well as platforms for drug testing.

## 3. Modeling of Viral Pathogenesis Using Adult Stem-Cell-Derived Organoid Systems

Due to the limitations of conventional cell line cultures and animal models, there has been a clear and pressing need for an advanced in vitro platform capable of recapitulating tissue physiology. Over the last few decades, there have been remarkable advances in establishing such systems, driven primarily by a developmental biologist, Yoshiki Sasai, and stem cell biologist, Hans Clevers, and their research teams. Both groups have developed self-organizing, three-dimensional in vitro culture systems that accurately recapitulate the physiology of the tissue of origin, such as the presence of multiple cell types (including stem cells), genetic stability, maintenance of regulatory pathways and tissue architecture [58]. Following the establishment of these organoid systems, multiple groups have adopted this technology both in basic science and translational research to address questions in fields such as host-pathogen interaction, tumor biology and disease modeling. In this part, we will concentrate on the application of adult stem-cell-derived organoid systems to study viral pathogenesis.

## 4. Uses of Adult Stem-Cell-Derived Organoid Systems in Modeling Viral Pathogenesis

Rotavirus is a leading cause of diarrheal disease in young children worldwide [59]. Every year there are hundreds of millions of cases, with hundreds of thousands of cases resulting in child death [59]. Oral vaccination against rotavirus has been effective in developed countries, but it only shows limited success in developing countries, with a continuing high rate of infection even amongst the vaccinated population [60]. An immortalized 2D cell culture system infected with a well-characterized laboratory strain has been widely adopted as the standard model to study rotavirus pathogenesis. However, a robust culture system for patient-derived strains is still needed in order to gain more information on the 110,000 strains of existing rotavirus. In 2015, two groups reported that 3D primary human intestinal organoid cultures could serve as such a platform [61,62]. Both laboratory and patient-derived strains showed rapid replication in human intestinal organoids within 24 h of inoculation. Yin et al. showed that the antiviral cytokine IFN-α as well as a VP7-targeted, neutralizing antibody and the nucleoside analog ribavirin, have virus-suppressive activity in the organoid system. Most interestingly, patient-derived strains grown in organoids showed differential sensitivity to IFN-α and ribavirin, suggesting that the organoid model system could be used in a personalized medicine setting to identify the most effective, strain-specific therapy for intervention. Saxena et al. demonstrated that rotavirus infection induces water influx to the lumen of intestinal organoids, mimicking rotavirus-induced diarrhea in vitro, and further demonstrated that the rotavirus enterotoxin NSP4 peptide also causes water influx in intestinal organoids. In addition to these two reports, Pan and colleagues have independently confirmed that the organoid system accurately mimics aspects of physiology during rotavirus infection and demonstrated the therapeutic use of different interferons [62]. Taken together, these reports showed further evidences that the human intestinal organoid system can serve as a faithful surrogate model system for studying viral pathogenesis.

Human noroviruses are another cause of acute gastroenteritis [63], and can lead to the death of young children, aged or immunocompromised individuals [64]. For many decades it was largely impossible to establish a norovirus culture system. In 2016, Ettayebi et al. reported the development of a system, based on a human intestinal organoid-derived epithelial monolayer, which could successfully cultivate norovirus [65]. Using this system, the authors found a strain-specific requirement for bile components and the FUT2 enzyme. As this organoid-derived system enables a robust reductionist approach, Ettayebi et al. showed that the bile acts on the target cell and not on the virus, and further that the bile is required only after the initial viral contact with the target cell. The authors also utilized human organoid lines isolated from people with different FUT2 genotypes and showed a strong requirement of FUT2 expression for GII.4 noroviruses, which faithfully recapitulates the epidemiologic data. Lastly, two sera containing neutralizing antibodies have been tested in the organoid model to validate their utility as an assay system. Altogether, this supports the use of intestinal organoids for the study of norovirus and susceptibility to viral infection.

Enteroviruses are single-stranded RNA viruses that commonly infect the gastrointestinal tract, but may also spread through the respiratory tract, and are transmitted mainly via the fecal-oral route. Enterovirus infection is predominantly without symptoms or causes moderate symptoms, similar to a mild cold, but in some cases leads to severe symptoms. In spite of the prevalence of enterovirus infection throughout the population, available knowledge about their pathology and mechanism of infection is very limited. In 2017, Coyne and colleagues demonstrated that the human intestinal organoid system is susceptible to infection by enterovirus, including echovirus 11 (E11), coxsackievirus B (CVB) and enterovirus 71 (EV71), making the system amenable to the study of these viruses [66]. Importantly, the authors also found that intestinal organoids retain cell-type-dependent susceptibility to virus infection and can induce antiviral and inflammatory responses following infection. In addition, Wolthers and colleagues identified that position 145 of the capsid protein VP1 of EV71 is a crucial determinant of viral infectivity by using human airway organoids [67]. The authors found that human airway organoids support EV71 replication in a strain-dependent manner, and that the replication kinetics depend on the amino acid present at position 145 of VP1.

Adenoviruses (AdVs) are common viruses and cause symptoms that range from mild and flu-like, such as sore throat, etc., to severe, such as pneumonia or diarrhea, and can even result in death. Vaccines are available against adenovirus type 4 and 7, which cause the most severe symptoms. Using mouse small intestinal organoids, Smith and colleagues elucidated the role of α-defensin, an antimicrobial molecule produced by Paneth cells, in infection by mouse adenovirus (MAdV) [68]. The authors generated mouse small intestinal organoids from either wild-type or *Mmp7*-deficient mice that lack functional α-defensin to compare the viral infection rate in intestinal organoids derived from these mice by monitoring GFP-tagged MAdV. It was found that the MAdV infection rate was greater in wild-type organoids than in *Mmp7*-deficient organoids, suggesting the functional importance of α-defensin in AdV infection. The authors have therefore proposed that AdVs might hijack the host immune system to increase viral infectivity. In the follow up study from the same group further demonstrated the power of organoid system as a proper culture platform to investigate adenovirus biology [69]. In this study, the authors applied human adenovirus (HAdV) on the human-derived intestinal organoid and revealed human intestinal organoid provide proper condition for adenovirus replication and sensitivity to interferon treatment. Moreover, an interesting observation came out from the study that HAdV-5p favorably infects goblet cells more than other cell types. This observation can be monitored in organoid systems since its cellular diversity differs from that of a conventional culture system.

Influenza A viruses (IAVs) have diverse subtypes classified based on the expression of the two surface molecules hemagglutinin (H) and neuraminidase (N). Due to the low fidelity of the viral RNA polymerase, various combinations of these two surface molecules are generated and define the host range of the virus from avian to mammalian species. Historically, several outbreaks of highly virulent IAV have been reported, including Spanish flu caused by the H1N1 subtype, Hong Kong flu by the H3N2 subtype, and Chinese flu by the H7N9 subtype [70,71]. Although new outbreaks occur continuously, it is impossible to predict which will result in a highly virulent subtype and therefore it is critical to establish an in vitro system that faithfully recapitulates in vivo pathophysiology. Yuen and colleagues reported an advanced method to generate 3D human airway organoids that harbor mature ciliated cells, thus accurately mimicking the relevant physiology of the human airway [72]. The authors applied their human airway organoid system to monitor virus infectivity with four different viral subtypes, H7N9/Ah, H7N2, H1N1pdm, and H1N1, which have different levels of infectivity in humans, as described in previous studies. The system successfully allowed viral replication of the H7N9/Ah and H1N1pdm subtypes but not the other subtypes, consistent with previous reports on the infectivity of these subtypes.

Respiratory syncytial virus is a single-stranded RNA virus infecting the airway. In most cases, RSV infection causes mild symptoms and infected people recover within a few weeks; however, it can also cause severe defects such as bronchiolitis and pneumonia in young infants, children, and the elderly. Due to the high risk of RSV infection, it has long been a challenge to develop a vaccine, a goal that was made yet more challenging due to the lack of a proper platform to study RSV. Clevers and colleagues reported the development of a human airway organoid system to model RSV infection [73]. The authors used human bronchoalveolar biopsies to establish airway organoids and confirmed that the system successfully recapitulates in vivo physiology by monitoring the organization and composition of cells in the organoids. In addition to establishing the airway organoid system, the authors demonstrated its potential application for studying host-virus interactions of RSV. Epithelial morphology changed dramatically upon infection with RSV, similar to the changes in morphology seen in vivo following RSV infection, and viral replication was prevented by pre-incubation with the RSV-cell fusion inhibitor, palivizumab. Interestingly, RSV infection caused an increased motility in infected cells, attributable to the NS2 molecule, further indicating that the airway organoid system faithfully recapitulates the in vivo physiology as a robust platform to study RSV-host interaction.

## 5. Applications of CRISPR/Cas9 Technologies in iPSC- and Adult Stem-Cell-Derived Systems for Viral Research

Genome editing based on CRISPR/Cas9 technology has been widely adopted across multiple fields of biomedical research. The CRISPR-Cas system is an adaptive immune system that defends bacteria and archaea against bacteriophages and pathogenic plasmids. CRISPR is a short, repeating ribonucleotide (crRNA) that functions to eliminate exogenous genetic elements by directing Cas endonuclease proteins [74]. The CRISPR locus is transcribed into a pre-CRISPR RNA transcript (pre-crRNA), which is then further processed into a mature crRNA. Trans-activating crRNA (tracrRNA) pairs with crRNA transcript repeat regions, and the crRNA and tracrRNA act together to guide the Cas9 enzyme to its target sites and activate nuclease activity against DNA complementary to the crRNA [75]. There are three distinct CRISPR-Cas systems classified by the way in which the pre-crRNA is processed [76], with the type II Cas9-based system being the most widely used for eukaryotic genome editing. Human ESCs and iPSCs are ideal systems for genetic manipulation because the selection of targeted clones based on drug resistance can be achieved without the loss of pluripotency. CRISPR/Cas9 technology has allowed the rapid and efficient manipulation of the genome in human stem cells due to its high specificity and effectiveness. In this part, we will summarize recent progress using CRISPR/Cas9 technology in human stem-cell-based systems.

## 6. Targeted Approaches to Identify Host Factors Regulating Infection

ZIKV exhibits a strong tropism toward neural stem cells over non-neural cells or neurons [18,41]. However, another closely related flavivirus, West Nile Virus (WNV), preferentially infects more differentiated neural cells [77]. Understanding the cell type-specific tropisms of viruses could be a key step in identifying the molecular mechanisms leading to infection, and eventually result in novel therapeutic targets for preventing viral infection. Single-cell transcriptome analysis of the human neocortex revealed the expression of several putative viral entry receptors, including AXL, which is expressed specifically in neural stem cells [78]. To test whether AXL is a critical ZIKV entry receptor in human neural stem cells, *AXL* was mutated in human pluripotent stem cells (hPSCs) by CRISPR/Cas9 genome editing. However, cerebral organoids derived from *AXL*-knockout hPSCs could still be infected efficiently and subsequently displayed microcephaly-related phenotypes, suggesting that AXL is dispensable for ZIKV infection in human neural stem cells [79]. Further loss-of-function analyses of other candidate entry receptors in the future will provide greater clarity regarding the mechanism of ZIKV tropism.

In addition to identifying the entry receptors themselves, CRISPR/Cas9 genome editing can be used to investigate regions of the entry receptor that are critical for infection. Chemokine receptor 5 (CCR5) is known to serve as a major coreceptor for HIV-1 entry into target cells [80]. Macrophages derived from *CCR5*-knockout iPSCs, which were generated by CRISPR/Cas9 genome editing, were resistant to infection by HIV [81]. A naturally occurring mutation of CCR5 (CCR5Δ32) was introduced into human iPSCs, and monocytes/macrophages derived from the genome-edited iPSCs were also resistant to infection by HIV [82]. The above are examples of approaches using targeted CRISPR/Cas9 genome editing to study host factors regulating viral infection and replication.

## 7. CRISPR/Cas9-Mediated Genome Editing in Adult Stem-Cell-Derived Organoid Systems

Following the establishment of CRISPR/Cas9-mediated genome editing in other systems, it has also been rapidly adopted in organoid culture systems in order to provide new insights into how genotypic differences result in physiological differences. The first application of the CRISPR/Cas9 technique to the organoid system demonstrated correction of the mutated CFTR gene in intestinal organoids derived from cystic fibrosis (CF) patients [83]. Several follow-up studies have subsequently been reported by other researchers that verify the feasibility of applying CRISPR/Cas9 technology to organoid systems [84,85,86,87,88].

Combining organoid systems with CRISPR/Cas9 genome editing broadens the applications of the organoid systems in many aspects. Firstly, the system facilitates monogenic disorder disease modeling (e.g., cystic fibrosis in intestinal organoids and alpha-1 antitrypsin deficiency in liver organoids), in addition to providing the translational potential to identify therapeutic interventions in those disorders [83,89]. These studies have now demonstrated that patient-derived organoids successfully mimic the pathophysiology of disease and, therefore, platforms such as CRISPR/Cas9 can be applied to investigate or ameliorate the readouts of disease at the genetic level in these systems. Secondly, the combination can provide a platform to study tumorigenesis by manipulating genes linked to cancer [85,88]. Several studies have shown that oncogenic driver mutations can be introduced into otherwise non-transformed organoids by CRISPR/Cas9 gene editing to model stepwise tumorigenesis. For example, Sato and colleagues delivered the oncogenic versions of well-known cancer-associated genes such as *APC*, *SMAD4*, *TP53*, *KRAS*, and *PIK3CA* into organoids and monitored tumor progression in xenografted mice [85,88]. The authors concluded that the mutations in well-known genes supply favorable conditions for tumor initiation, but that further mutations are required to induce the metastatic phenomenon. This was confirmed independently in the work by Drost et al. [85]. Thirdly, advanced use of CRISPR/Cas9 technology to mediate multiple gene knockouts in parallel in organoids allows loss of function studies with paralogous genes, in which redundancy between paralogues might normally prevent a phenotype becoming penetrant when a single paralogue is knocked out [90]. In addition, a novel method for generating conditional knockout alleles in organoids has been developed for research in this area [91]. Taken together, recent advances in manipulating organoid systems with CRISPR/Cas9 technology have opened enticing potential new avenues in biomedical research. However, although simple genetic alteration with CRISPR/Cas9 technology has been widely adopted, genome-wide screening with CRISPR/Cas9 in organoids has not yet been reported, representing one area in which further development is required to realize the full potential of these systems. Obvious technical challenges include the necessity of specifically modifying the stem cells present in organoids to establish stable phenotypes and that scaling up the culture size is difficult when compared to conventional 2D cell lines and iPSC lines. Once these barriers are overcome, however, such a platform will open up the possibility of performing forward genetic screens in organoids for the identification of, for example, novel cancer drivers or genes required for viral infection. Moreover, CRISPR/Cas9-mediated gene editing on the organoid system will extend not only basic understanding of host-virus interaction but also shed light on the pre-clinical potential and possibility of personalized medicine in the near future.

## 8. Applications of Genome-Wide CRISPR/Cas9 Screening

In addition to targeted approaches, genome-wide CRISPR screening is a powerful tool to identify crucial host restriction and dependency factors in a non-biased manner. Loss-of-function screens can be used to assess the impact on viral infection upon knockdown of individual host genes. Although initial attempts with RNAi-based screening have provided valuable insights [92], this technology is often hampered by partial depletion of the target or silencing of knockdown effects. The advent of CRISPR/Cas9 genome editing has revolutionized the field of mammalian pooled genetic screening [93] through the ease with which the system can be multiplexed. Multiple CRISPR sgRNA libraries, which enable the complete disruption of gene expression on a genome-wide scale, are now widely available [94] and there have been several cases of genome-wide knockout screens performed to identify host-virus interactions that have been successful. For example, screens have been performed to identify the host factors required for the replication of flaviviruses, such as ZIKV, Dengue virus (DENV) and WNV [95,96]. These studies found that multiple host factors involved in endocytosis and transmembrane protein processing, including the endoplasmic reticulum membrane complex, are important for flavivirus replication. A similar approach for HCV infection revealed critical factors including RNA-binding proteins and enzymes involved in metabolism, suggesting that, in spite of common replication strategies, different flaviviruses may rely on divergent molecular pathways for productive infection [97]. Another CRISPR/Cas9 screen focused on WNV infection identified essential host genes responsible for WNV-induced cell death, of which multiple are found in the ER-associated protein degradation (ERAD) pathway [98]. Interestingly, genes associated with ERAD are not important for WNV replication, demonstrating the effectiveness of CRISPR/Cas9 screening in revealing downstream host effectors for virus-mediated cytotoxicity. Yet another study identified host factors required for HIV infection but not for cellular proliferation and viability, which could be ideal target pathways for therapeutic intervention [99]. These studies exemplify the utility of CRISPR/Cas9 screening in providing a system-wide view of human-virus interactions and in revealing potential therapeutic targets to treat human viral diseases. So far, there are limited examples to apply genome-wide CRISPR/Cas9 screening to stem-cell-derived systems yet. However, considering the robustness of currently developed screening toolsets, stem-cell-derived viral disease relevant cell types, such as neural progenitor cells for ZIKV research, would be attractive targets for future genome-wide CRISPR/Cas9 screening.

## 9. Conclusions and Future Perspectives

The main contribution of the development of advanced systems such as iPSCs, organoids, and CRISPR/Cas9 genome editing is providing the right platform for researchers to study in vivo-like physiology in a dish. Both iPSC and organoid systems currently have their strengths and limitations, which makes their use challenging but attractive. iPSCs, especially patient-derived iPSCs, have great potential for disease modeling, such as the viral infection modeling, as exemplified with ZIKV research. In combination with genome-wide CRISPR screening, the high-throughput identification of genetic factors that influence virus susceptibility and the validation of therapeutic potential will soon be possible. In addition, the directed differentiation of iPSCs can also provide insights into how viral infection causes pathophysiology during developmental processes. However, there are still technical limitations that curb its potential application in some areas of research.

Because adult stem-cell-derived organoid culture systems recapitulate complex aspects of in vivo physiology in vitro, they are excellent for the reductionist study of host-virus interactions. Whilst conventional 2D in vitro cell line culture and studies using model organisms have contributed greatly to our understanding of the basic biology of infection and have allowed the identification of host defense and escape mechanisms for diverse pathogens and the development of antiviral therapies, conventional approaches are limited by the lack of appropriate target cells and physiology at the level of the tissue. Thus, the development of a system that faithfully mimics the original environment, such as with adult stem cell organoids, has been eagerly anticipated in the field for some time. During the last decade, a rapid expansion of organoid technology has enabled researchers to study host-pathogen interactions in a more physiologically relevant setting. Organoid technologies will continue to broaden our knowledge of infection, cell tropism, and mechanisms of interaction with the host by viruses that were difficult to study in the past. Nevertheless, the system could be further optimized, since it still lacks certain components present in vivo, such as immune cells, stromal cells, and blood vessel architecture. To address these issues, researchers are testing diverse co-culture systems, with a particular focus on the inclusion of immune cells [100,101].

CRISPR/Cas9-mediated genome editing is now widely applied to manipulate host genomes and conduct high-throughput forward genetic screens on a genome-wide scale. However, the CRISPR/Cas9 system still involves potential risks and difficulties such as off-target effect, difficulties on a delivery method to organoid, validation of the process, etc. [102,103]. To overcome such difficulties, numerous technical attempts improved the efficiency and accuracy of the system, including generating Cas9 variants that produce significantly fewer off-target effects [104,105,106], developing and improving diverse material delivery methods [83,107,108,109]. Therefore, such applications will be widely employed to dissect the mechanisms of host-pathogen interactions. Knockout of a specific gene and/or the substitution of an amino acid at the desired position by CRISPR/Cas9 has become routine in biomedical research, even in advanced cell culture systems, such as organoid systems. Moreover, many studies have now performed genome-wide CRISPR screens on cultured cell lines, including iPSCs, and even in vivo in animal models, suggesting great potential for this platform in a wide range of applications. We expect that, in the near future, this will become a routine tool for studies involving organoid systems.

In summary, numerous challenges have recently been surmounted in the drive to develop more accurate, more complex, and more robust platforms to investigate biology of host-virus interactions in infectious diseases. We now have access to advanced systems for disease modeling, such as iPSCs, organoids, and CRISPR/Cas9 genome editing tools. Altogether, these new developments promise an acceleration in the progress of our biomedical research into human disease, including in the field of viral disease.
